# Polymer-assisted self-assembly of gold nanoparticle monolayers and their dynamical switching†
†Electronic supplementary information (ESI) available: Reflection spectra of AuNP monolayer at the LLI, and repeatability; reflection spectra of different packing densities; scheme showing how the AuNPs actuate on the Si substrate. See DOI: 10.1039/c6nr05199e. The dataset of the figures in this paper can be found at http://dx.doi.org/10.17863/CAM.1194



**DOI:** 10.1039/c6nr05199e

**Published:** 2016-08-10

**Authors:** Tao Ding, Adam W. Rudrum, Lars O. Herrmann, Vladimir Turek, Jeremy J. Baumberg

**Affiliations:** a Nanophotonics Centre , Cavendish Laboratory , University of Cambridge , CB3 0HE , UK . Email: dt413@cam.ac.uk ; Email: jjb12@cam.ac.uk

## Abstract

Dynamic switching of plasmonic monolayers built of gold nanoparticles (AuNPs) is achieved using nano-coatings of poly(isopropyl acrylamide) (PNIPAM). The distance between AuNPs can be dynamically tuned through the repeatable expansion and contraction of the PNIPAM shells at different temperatures, which results in rapid switching of the optical properties of the AuNP monolayer.

## Abstract 2

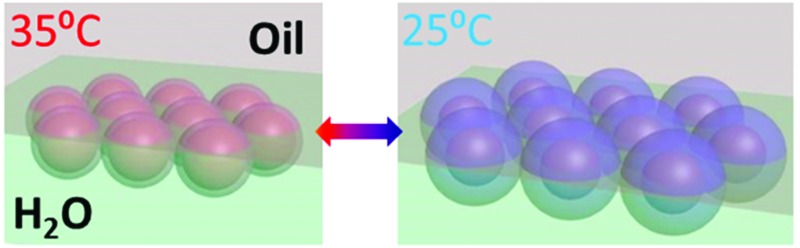
Dynamic switching of plasmonic monolayers built of gold nanoparticles (AuNPs) is achieved using nano-coatings of poly(isopropyl acrylamide) (PNIPAM).

## Introduction

The topic of tuneable plasmonics has sparked great interest in recent years^1–6^ as it offers realistic hopes for plasmonically active thin films that enable a wide range of applications from variable focus mirrors to biosensing processes such as surface plasmon enhanced Raman spectroscopy and catalysis.^7–10^ However, the fabrication and actuation of ordered thin films of nanoparticles such as gold (AuNPs) remains challenging.^11^ Liquid–liquid interfaces (LLIs) are attractive as a scaffold for the self-assembly of AuNP monolayers, because they are self-healing and exhibit surface energies that can be reliably controlled. Because of the fluidic nature of the interface, the distance between the NPs can be tuned *via* their surface chemistry,^12,13^ by changing the pH of the solvents^13^ or by changing the ratio of NPs to interfacial area.^13–15^ The issue with these previous methods of adjusting the distance between particles is that they are nearly invariably irreversible, the spectral tuning range is small (<50 nm) and any reversible tuning can only be achieved by (very slowly) changing the chemical environment of one of the phases – tuning the interparticle separation has not been shown with a truly external stimulus such as temperature or light, and has never been rapid and reversible at LLIs.^16,17^ Here we present a route to assembling active NPs into monolayers, and show how their spacing can be rapidly tuned to change their colour.

The application of the temperature-responsive polymer PNIPAM (poly-*N*-isopropylacrylamide) to adjust the spacing of AuNPs has been demonstrated previously.^18–25^ The reversible water coordination of this polymer contracts its volume strongly above a transition temperature of 32 °C when it becomes hydrophobic, reversing on cooling as the water resolvates the polymer. However, since the PNIPAM is mostly grown as thick films (>50 nm) on the surface of AuNPs^18–22^ or the Au NPs are randomly attached to large globular microgels of PNIPAM,^23–26^ temperature-induced plasmon resonance shifts remain small (Δ*λ* < 20 nm), slow, and imperceptible in colour appearance. While there are some reports that PNIPAM can attach to Au NPs *via* ligand exchange, so far this has yielded mixtures of non-uniform aggregates or clusters,^27,28^ which give few insights into the potential for defined plasmonic structures.

In this communication, we report the preparation of AuNP@PNIPAM NPs through a ligand exchange method. The PNIPAM shells here not only facilitate the transfer of 15 nm diameter Au NPs to the interface between water and hexadecane but also serve as a temperature-controlled spacer between the AuNPs, thereby creating a dynamically tunable plasmonic AuNP monolayer at the interface. By changing the packing density of the AuNPs at LLI, the plasmon wavelength shift can approach 100 nm.

## Experimental

**Fig. 1:**
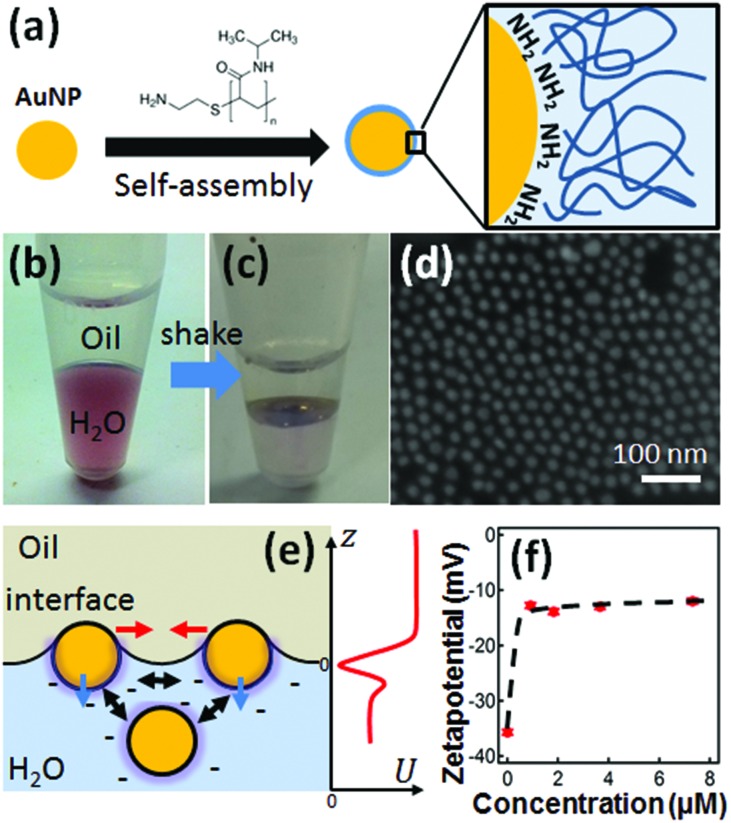
Formation of AuNP monolayer at the liquid–liquid interface (LLI). (a) Scheme of PNIPAM-NH_2_ self-assembly on AuNP surfaces to form AuNP@PNIPAM core shell structure. (b, c) Photos of AuNP sample before (b) and after (c) agitating the two phases of AuNP@PNIPAM-H_2_O and hexadecane. (d) SEM image of Au NP monolayer transferred onto Si wafer. (e) Schematic illustration of assembly at LLI showing forces: capillary and van der Waals attraction (red), electrostatic and steric repulsion (black), solvation and line tension (blue), red curve (right) indicates surface potential near the LLI. (f) Zetapotential of AuNP@PNIPAM with increasing concentration of PNIPAM.

Standard AuNP (15 nm) dispersion was obtained from BBI in concentrations of 7 × 10^11^ particles per mL, which possess excess citrate capping for charge stabilisation. An amount of 10 mg of PNIPAM terminated with amino groups (*M*_n_ = 5500, Sigma Aldrich) is dissolved in 1 mL of H_2_O. The zetapotential of the AuNPs is measured with a Nanosizer (Malvern). The PNIPAM assembly onto the AuNP surfaces is carried out initially by mixing in aqueous phase. After reaching equilibrium in a few minutes, these NPs are moved to a bi-phase system where hexadecane is added (Fig. 1b). During vigorous shaking of this two phase system, liquid bubbles are generated. The pH of the aqueous phase is around 6. The droplets formed quickly move to the interfacial region (with surface area of ∼2.8 × 10^–5^ m^2^). After being given time to settle, the AuNPs then form a layer at the interface (Fig. 1c).

Once the layers were assembled they were studied with reflection spectroscopy. An optic fibre was clamped in place above the samples to both illuminate the sample and collect the reflected light. It is necessary to remove some of the organic phase from the samples, leaving only a thin layer to ensure the interface remains intact. This allowed for the optical fibre to be lowered closer towards the interface. In order to vary temperature, samples were placed in a container of water on top of a hot plate to create a heating bath whose temperature was calibrated with thermometer.

As for the dark field scattering spectra measurement, the Au NPs monolayer were formed at LLI within a glass container under the dark field microscope. The glass container is heated with Linkam hot stage to vary the temperature of the liquids. The spectra were collected through an optical fiber coupled spectrometer (QE65000, Ocean Optics) while cycling the temperature.

The samples at the interface are transferred to a Si substrate *via* standard dip-coating. A plasma-cleaned silicon wafer is inserted from an angle below the LLI and then slowly lifted to capture the monolayer away from the interface allowing it to then dry on the Si substrate. The response of the AuNP monolayer is also monitored with a dark field microscope and the scattering spectra were recorded as the temperature cycles. A scanning electron microscope (SEM, LEO 1530VP) is used to characterize the morphology of the Au monolayer.

Finite-difference time-domain simulations (FDTD, Lumerical Solutions, Inc.) were carried out under plane wave illumination of a hexagonally packed monolayer of spherical AuNPs placed at the interface of a hexadecane (*n* = 1.43) and a water (*n* = 1.33) sheet. Periodic boundary conditions were applied to the side of the hcp unit cell. The additional reflectivity originating from the air-hexadecane layer was also accounted for. Similarly, scattering simulations were performed based on the total-field/scattered-field formalism. The dielectric function of gold was taken from Johnson and Christy.^29^

## Results and discussion

The amino-terminated PNIPAM (PNIPAM-NH_2_, Experimental) forms a self-assembled monolayer on 15 nm AuNPs *via* coordination bonding between Au and –NH_2_ (Fig. 1a). This self-assembly process is accelerated through several cycles of heating and cooling, bringing PNIPAM to thermodynamic equilibration with the aqueous phase.^30^ The resulting coated particles along with water are mixed with hexadecane through vigorous shaking and settle at the H_2_O/hexadecane interface (Fig. 1b and c) within 10 min. This method also works for different types of NPs, such as AgNPs (Fig. S1†). Other nonpolar organic solvents such as heptane and FC-40 are also observed to support such AuNPs monolayers. This system works well for AuNPs smaller than 20 nm, while for AuNPs 60 nm or larger, no noticeable monolayer of AuNPs can be formed at the LLI due to the high barrier from strong electrostatic repulsion between AuNPs and the charged interface.^31^ One role of PNIPAM in the system is to further reduce the surface charge by stripping off citrate ligands from each AuNP (Fig. 1f) while maintaining their stability through steric hindrance. The other role of PNIPAM is to reduce the surface tension of water which modifies the interfacial energy of AuNPs at the LLI.^32^ These two effects aid NP assembly at the interface which is more challenging when using the original citrate-stabilized AuNPs. The net result of the PNIPAM coating is that the potential barrier becomes sufficiently weak to be overcome during the process of agitation. Droplets form with NPs trapped at their surfaces, collect at the interface, and burst leaving behind a single monolayer at the LLI. This is a similar process to Girault's work^33^ but with PNIPAM as the emulsifier. The advantage of using PNIPAM here is to enable dynamic tuning of the separation between the AuNPs which leads to switchable plasmons.

The AuNP monolayer is manually transferred onto a clean silicon substrate (pretreated with oxygen plasma) to allow SEM characterization. The SEM image (Fig. 1d) reveals a near-hexagonal local arrangement of the AuNPs with few large-scale defects in the monolayer (though drying is highly likely to significantly alter the structure of the monolayer). We stress however that such SEM images cannot indicate the real morphology of the AuNP monolayer at the LLI since removing both solvents in vacuum modifies the films. It does however give some indication of their topology (as we discuss below, optical spectra are needed to estimate the particle separation at the LLI). Monolayer organization at the LLI is favored with the NPs tightly and vertically confined by the stabilization of the interface (the sharp interface potential well prevents bilayers) and laterally balanced by electrostatic, capillary and van der Waals forces^34^ which work in complex interplay with the steric repulsion (Fig. 1e). The AuNP@PNIPAM monolayer at the interface is thermodynamically stable.

**Fig. 2:**
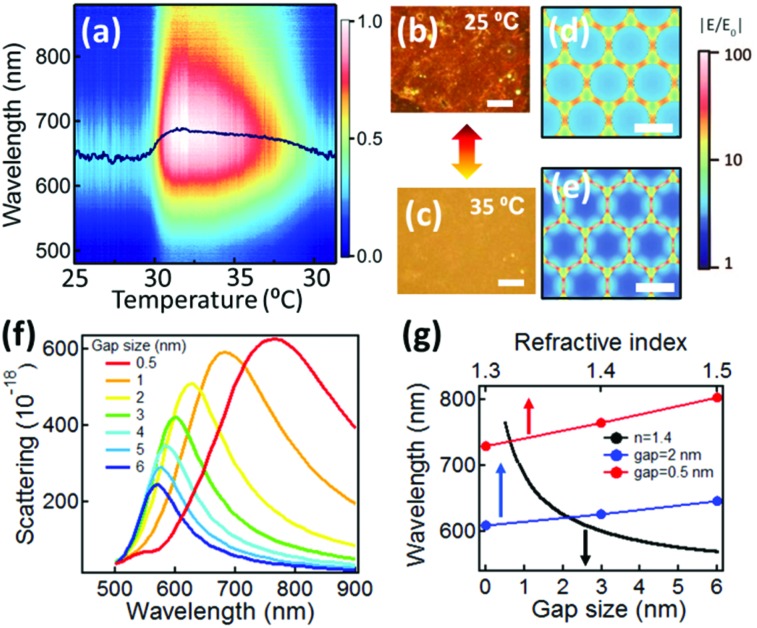
Scattering spectra of AuNP monolayer at the LLI (2D NP density 2000 μm^–2^) and simulations. (a) Scattering spectra of AuNP monolayer as temperature cycled between 25 °C and 35 °C. Black line is fitted peak wavelength. (b, c) Dark field images of the AuNP monolayer at (b) 25 °C and (c) 35 °C, scale bars 5 μm. (d, e) Simulated electric field enhancement of periodic Au NP monolayer for gap sizes of (d) 2 nm and (e) 0.5 nm. Scale bars: 15 nm. (f) Simulated scattering spectra of AuNP monolayer for gap sizes from 0.5 to 6 nm. (g) Resonance spectral shift as a function of gap size (with *n* = 1.4) and refractive index (with *d* = 0.5 and 2 nm).

The AuNP@PNIPAM monolayer formed at the LLI is thus found to be stable and active for weeks as long as the LLI remains. For optical measurements, the volume of organic phase is reduced to enable us to approach close to the monolayer with short-working-distance microscope objectives and optical fibers. Scattering spectra (Fig. 2a) are recorded while cycling the temperature through the PNIPAM phase transition temperature (*T*_c_ ∼ 32 °C). As the temperature increases from 25 °C to 35 °C, the scattering color of the monolayer changes from red to yellow (Fig. 2b and c) and the scattering red-shifts from 640 to 690 nm, broadening strongly (Fig. 2a). When the temperature cools back down to 25 °C, the scattering colour of the AuNP@PNIPAM monolayer recovers to the initial state (red) and the scattering spectra blue-shift back to 641 nm (Fig. 2a). The dynamic measurements clearly show that both red- and blue-shifts trigger when the temperature hits the PNIPAM hydrophilic–hydrophobic transition temperature, *T*_c_, and thus confirm that contraction and expansion of the PNIPAM switches the plasmonic response. We also believe the free PNIPAM remaining in aqueous phase (∼90 μg) remains in dynamic equilibrium with the AuNP monolayer at the LLI, as the temperature cycles through the transition temperature. The whole process of switching is reversible and repeatable for many cycles (Fig. S2†). Since the refractive index changes of PNIPAM when hot (1.41) and cold (1.35) are less than 5%,^35^ the plasmon shift contributed by this factor is typically smaller than 10 nm.^36^ This is further confirmed from simulations (Fig. 2g) that show the plasmon shift of arrays with either small- (red line) or large-interparticle gaps (blue line) due to changes of the surrounding refractive index are always smaller than 15 nm. Therefore, the major contribution to the large plasmon shifts observed (>50 nm) is dominated by the change of AuNP separation. Finite-difference time-domain (FDTD) simulations based on a periodic 2D plasmonic crystal model show the field distribution within the AuNP arrays (Fig. 2d and e) with increasing concentration of field between the AuNPs as the gap size decreases from 2 nm (Fig. 2e) to 0.5 nm (Fig. 2d). In this type of coupled-plasmon mat, it is the particle distances which dominate the resonance wavelength rather than the precise local ordering (periodic or amorphous).^37,38^ The simulated scattering spectra gradually red-shift as the separation between the AuNPs reduces from 6 to 0.5 nm (Fig. 2f). Comparison with our experimental spectra (Fig. 2a) thus provides an estimate of the lateral distance changes between AuNPs in the monolayer, from 1.6 nm down to 0.9 nm (black line in Fig. 2g).

The optical properties of the AuNP@PNIPAM monolayer are further characterized using bright field microscopy and reflection spectroscopy (Fig. S3a and b†). These show similar temperature-dependent spectral shifts (between 640 to 670 nm for *T* = 30 to 35 °C) compared to the scattering spectra (Fig. 2a). Simulations of the reflection spectra (Fig. S3c and d†) also consistently imply the distance between AuNPs in the monolayer decreases from 1.7 to 1.1 nm when heated, increasing back to 1.7 nm when cooled back to 30 °C. Thus our spectroscopy robustly identifies the AuNP spacing, showing a two-fold variation (as expected from such PNIPAM layers) is possible to achieve in the LLI monolayer, with reversibility over at least 10 cycles. Separate measurements have shown the capability for switching such PNIPAM AuNP constructs on microsecond timescales.^30,39^

The interparticle distance is also controlled by the number of particles confined to the LLI per unit area. The interface area is controlled by the container diameter (6 mm) with the packing density defined by the volume of AuNPs which self-assemble into it. We increase the volume of AuNP solution while keeping the total volume fixed at 0.3 mL, so that the packing density of the AuNPs increases at the LLI (Fig. S4a†). This yields corresponding reflection spectra showing a gradual red-shift from 589 to 651 nm (Fig. S4b†). This is due to the compressed distance between AuNPs when more are transferred to LLIs of the same area. Increasing the AuNP density further results in a broader peak beyond 600 nm (Fig. S4b†) and the loss in thermal tuneability of the plasmon mat.

**Fig. 3:**
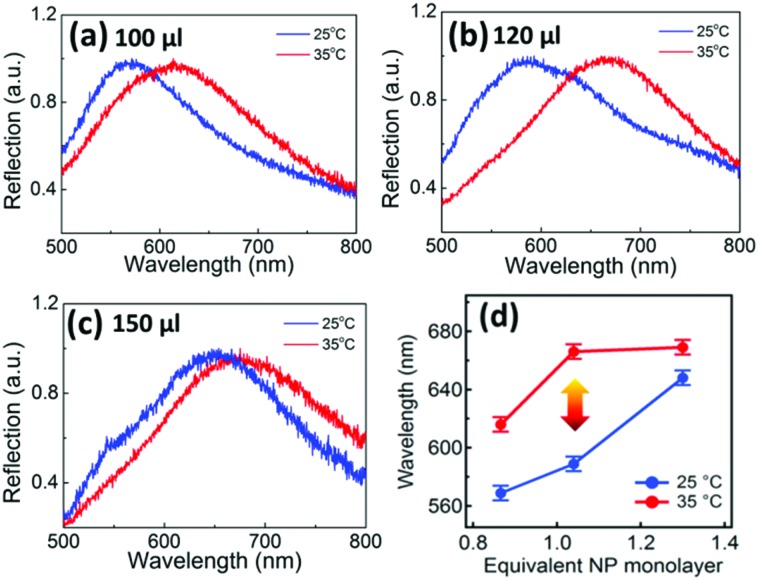
(a–c) Reflection spectra at 25 °C and 35 °C of AuNP monolayers at the LLI with different packing density. Estimated equivalent AuNP monolayer at interface (assuming all AuNPs move there): (a) 0.866, (b) 1.04, and (c) 1.3 (corresponding to 100, 120 and 150 μl of AuNP solution). (d) Change in reflection peak wavelength for increasing AuNP coverage, extracted from (a–c), in terms of equivalent monolayer coverage of the NPs at the LLI.

The AuNP monolayers with different packing density deliver different shifts of the plasmon resonance under the same temperature cycling range (Fig. 3). As noted above, at room temperature the reflection spectra of the AuNP monolayers red-shift with increasing packing density (Fig. 3a–c, blue curves). When the temperature increases to 35 °C (Fig. 3a–c, red curves), the reflection peaks red-shift by 46, 80, and 22 nm respectively on heating above *T*_c_, summarized in Fig. 3d. As soon as the coverage approaches a full equivalent NP monolayer (where the estimated coverage assumes a negligible PNIPAM thickness, see ESI† AuNP packing density calculation), the red shift in the peak wavelength of the contracted state saturates. At this point, the phase-transition-induced spectral shift is largest (Δ*λ* = 80 nm) which corresponds to a packing density of 3000 μm^–2^ (120 μL of AuNP solution added, giving a full monolayer at the interface). This optimal packing density keeps the NPs far enough apart when the PNIPAM is swollen to largely decouple plasmonically, but provides enough pre-strain to rapidly collapse their spacing when the water is expelled above the phase transition. Higher densities give more pre-strain but pre-compress the soft PNIPAM shells, giving already a strong red shift when cold. Smaller densities possess too little pre-strain to drive the NPs together.

**Fig. 4:**
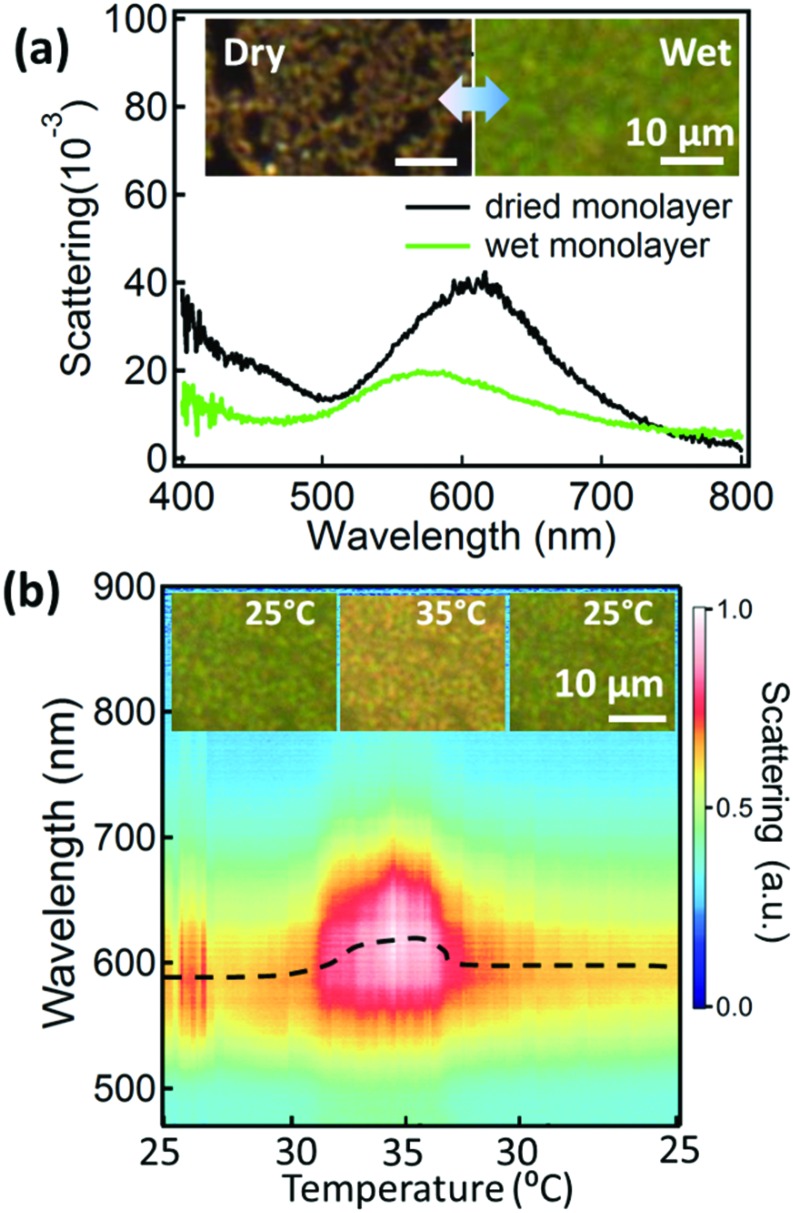
Scattering of AuNP monolayer on Si substrate. (a) Scattering spectra of dry and wet AuNP monolayer. Insets are dark field images of monolayer in dry (left) and wet (right) states, scale bars 10 μm. (b) Temperature cycled scattering spectra of wet AuNP monolayer on Si substrate. Black line gives fitted peak wavelength. Insets show dark field images at 25 °C (left), 35 °C (middle) and back to 25 °C (right). Scale bars are 10 μm.

Although the LLI provides a soft and flexible environment for the AuNPs to move easily within, it is not a convenient medium for device fabrication or many practical applications. Therefore, we also transfer the AuNP@PNIPAM monolayers onto Si substrates (as above) and investigate the tuneability of these supported monolayers. The dried AuNP@PNIPAM monolayer shows a scattering peak at 610 nm (Fig. 4a, black curve). Upon wetting, the scattering peak blue-shifts to 568 nm (Fig. 4a, green curve) mainly because the whole film expands when the PNIPAM swells with water, increasing the distance between AuNPs (Fig. S5†). The contribution from refractive index change is minor here dominated only by the standard red-shift after wetting the film. Dark field images (Fig. 4a, left inset) show that the dried AuNP@PNIPAM monolayer on the substrate presents many cracks (with the PNIPAM collapsed onto the Au surface), appearing orange in scattering, while the wet monolayer appears more uniform and green (Fig. 4a, right inset). The change of this substrate-supported hydrated AuNP@PNIPAM monolayer is monitored with scattering spectra while again cycling the temperature between 25 °C and 35 °C (Fig. 4b). Dark field microscopy images show reversible changes of color between green and yellow (Fig. 4b inset). The spectral shifts of the AuNP@PNIPAM monolayer on the substrate show similar trends to the monolayer at the LLI (Fig. 2a) but with smaller shifts (Δ*λ* = 35 nm on the Si substrate *vs*. 50 nm at the LLI), which could be due to the reduced pre-strain from stronger Van de Waals interactions on the Si substrate. Nevertheless, it confirms that the AuNP@PNIPAM monolayer remains temperature active even after transfer onto a solid substrate which therefore has potential for device applications.

## Conclusions

In summary, we fabricated temperature-tunable plasmonic monolayers of AuNPs at the LLI using coating with PNIPAM-NH_2_. The self-assembly has two component processes, one being the decrease in zetapotential from attachment of amine-terminated PNIPAM (Fig. 1f), which allows the AuNPs to easily absorb at the LLI. The second is the reorganisation of these AuNP@PNIPAM particles at the LLI through interparticle interactions. More detailed quantitative models of assembly at the LLI are currently not available for such systems due to the difficulty of solving fully the charge distribution near such a liquid interface with nanoparticles, although this is highly desirable. Because of the thermal responsivity of PNIPAM, the distance between AuNPs can be dynamically tuned with small changes in temperature around *T*_c_, which results in reversible switching of the optical properties of the AuNP monolayer. Simulation results match the experimental spectra, implying the distance between AuNPs increases from ∼0.9 ± 0.1 nm when hot to ∼1.6 ± 0.1 nm when cool. Such 2D plasmonic mats can be transferred to substrates without losing their tunability, holding great potential for various functional applications, such as temperature sensors, tunable SERS, and color changing displays. The polymer-assisted formation of close-packed AuNP monolayers also provides new pathways for 2D-confined crystallization of nanoparticles, which can be exploited for more complex geometries.

## Author contribution

TD conceived the idea and designed the experiments. AWR and TD performed AuNP monolayer preparation, spectra and SEM characterizations. LOH performed FDTD simulations. TD, LOH, VT and JJB discussed and analyzed the data and contributed to the writing of the manuscript.
