# Supplementary material for: Polymer-assisted self-assembly of gold nanoparticle monolayers and their dynamical switching
Source: Nanoscale. 2016 Aug 10;8(35):15864–9. doi: 10.1039/c6nr05199e (PMC5166565; doi:10.1039/c6nr05199e)
Supplement: Supplementary file 1 [file NR-008-C6NR05199E-s001.pdf]

Electronic Supplementary Material (ESI) for Nanoscale.
This journal is © The Royal Society of Chemistry 2016

Supplementary information

Polymer-assisted self-assembly of gold nanoparticle monolayers and their dynamical switching

Tao Ding*, Adam W. Rudrum, Lars O. Herrmann, Vladimir Turek, and Jeremy J. Baumberg*

Nanophotonics Centre, Cavendish Laboratory, University of Cambridge, CB3 0HE, UK

Email: dt413@cam.ac.uk (TD); jjb12@cam.ac.uk (JJB)

Au NP density calculation

The number of Au NPs (which are used as provided from BBI) per milliliter is estimated as N~10^{11}. For a volume V of d=20 nm diameter Au NPs added for assembly at the LLI, and assuming that all the Au NPs transfer to the LLI (more than 99% is confirmed from spectroscopy of the solution), the average packing density of Au NPs at the LLI is $\phi=4NV/(\pi D^2)$, where D is the diameter of the LLI inside the container. This monolayer estimate is used to provide the *x*-axis scale in Fig.3(d).

If the PNIPAM shell is also considered, the area each Au@PNIPAM takes up at the LLI is increased by $(1+2l/d)^2$, and the the packing density decreases by the same ratio. For a typical PNIAPM globule size of l~10 nm for this molecular weight PNIPAM, this would be 75% smaller compared to the close-packed Au NP monolayer with no coating. However it is likely that the PNIPAM can be much more compressed than this, given that we see the maximum spectral change near the expected close-packed AuNP monolayer coverage (Fig.3d).

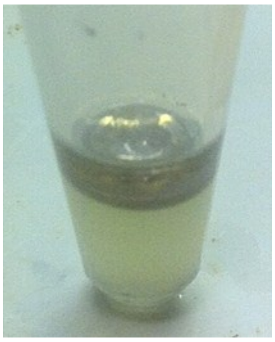

Figure S1. Photo of Ag NP monolayer at the LLI formed using the same method as for the AuNP monolayer shown in Figure 1c.

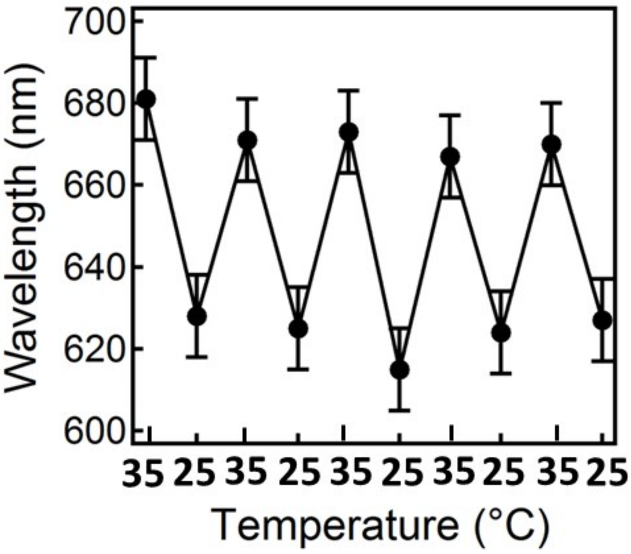

Figure S2. Change of reflection spectra peak of AuNPs monolayers at LLI with 5 cycles of heating and cooling between temperature of 25 °C and 35 °C.

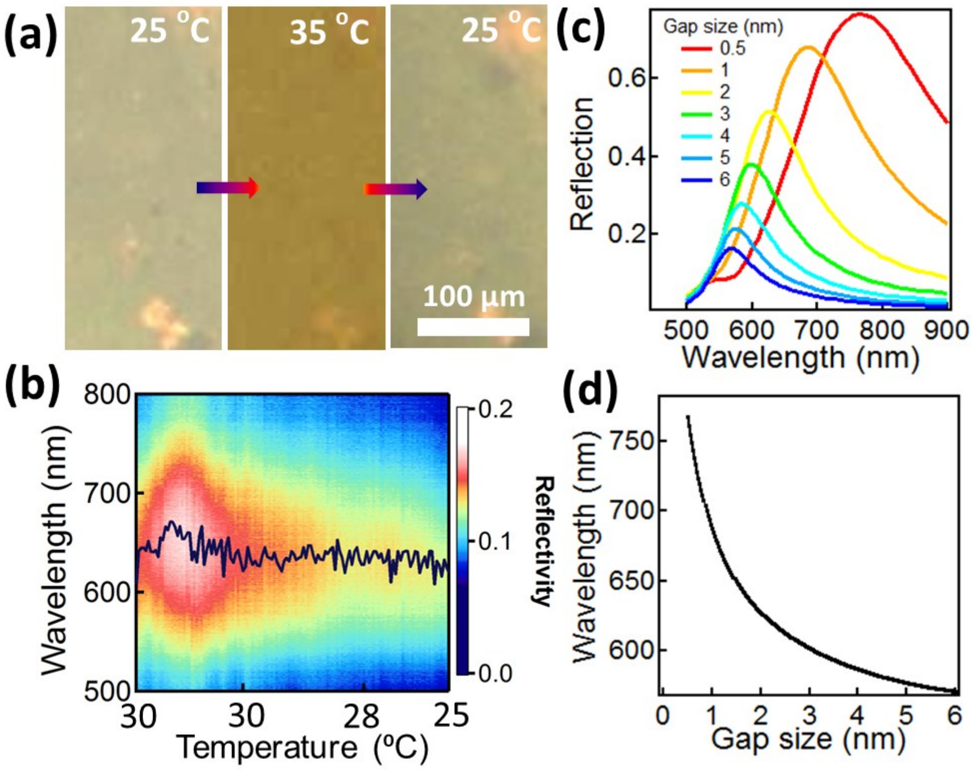

Figure S3. Reflection spectra of AuNP monolayer at LLI and simulations. (a) Bright field image of an AuNP monolayer at LLI with the change of temperature at 25°C, 35°C and back to 25°C. (b) Reflection spectra of an AuNP monolayer as the temperature increases from 30°C to 35°C and then decreases to 25°C. (c) FDTD simulations of the reflection spectra of an AuNP monolayer at LLI with different gap sizes. (d) Change of the wavelength of the reflection peak as a function of gap size.

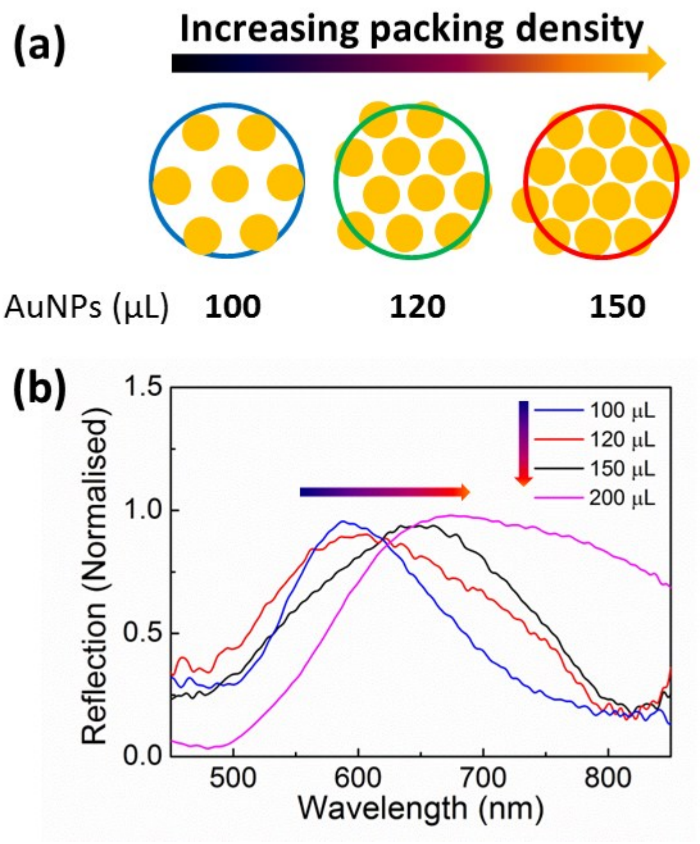

Figure S4. (a) Scheme of Au NP monolayer illustrating the growth in packing density as the amount of added AuNP solution is increased. (b) Reflection spectra of AuNP monolayers (25°C) for different amounts of AuNP added.

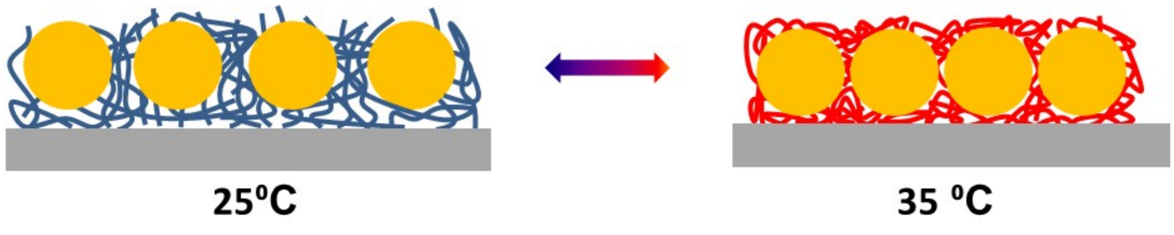

Figure S5. Schematic illustration of the actuation of AuNPs/PNIPAM monolayer on Si substrate.
